# An Unusual Presentation of Type B Aortic Dissection as Out-of-Hospital Cardiac Arrest Complicated by Spinal and Renal Ischaemia Along With Atrial Fibrillation, Stroke, and Severe Stenosis in Obtuse Marginal Branch: A Therapeutic Dilemma

**DOI:** 10.7759/cureus.26011

**Published:** 2022-06-16

**Authors:** Zahid Khan, George Besis, Yousif Yousif, Animesh Gupta

**Affiliations:** 1 Acute Medicine, Mid and South Essex NHS Foundation Trust, Southend-on-Sea, GBR; 2 Cardiology and General Medicine, Barking, Havering and Redbridge University Hospitals NHS Trust, London, GBR; 3 Cardiology, Royal Free Hospital, London, GBR; 4 Internal Medicine, Barking, Havering and Redbridge University Hospitals NHS Trust, London, GBR; 5 Acute Internal Medicine, Southend University Hospital NHS Foundation Trust, Southend-on-Sea, GBR; 6 Acute Internal Medicine/Intensive Care, Barking, Havering and Redbridge University Hospitals NHS Trust, London, GBR

**Keywords:** recurrent aspiration pneumonia, hypoxic ischemic brain injury, out-of-hospital cardiac arrest, tracheostomy placement, hypertension and therapy, renal artery occlusion, st-elevation myocardial infarction (stemi), type b acute aortic dissection, types of aortic dissection

## Abstract

Aortic dissection (AD) is a catastrophic cardiovascular problem that can be challenging to diagnose sometimes. Despite diagnostic challenges, it requires a high degree of suspicion and prompt treatment is vital to its successful management. AD can be divided into type A aortic dissection (TAAD) and type B aortic dissection (TBAD). TAAD is characterised by dissection in the ascending aorta whereas TBAD does not have dissection in the ascending aorta. TBAD is usually managed conservatively, and patients receive medical therapy such as antihypertensive medications, analgesia, and rehabilitation. This, however, is complicated by malperfusion of certain organs, which can be life-threatening. Patients who have malperfusion of certain organs should be managed aggressively and endovascular aortic repair should be considered in such cases. We present a case of a 63-year-old patient who presented with out-of-hospital pulseless electrical activity cardiac arrest and was successfully resuscitated. An electrocardiogram showed new-onset atrial fibrillation with ST-segment depression and a coronary angiogram showed severe stenosis in the obtuse marginal branch of the left circumflex artery. A computed tomography scan of the thorax and abdomen showed TBAD with an occluded right renal artery and the patient was conservatively managed. The patient was discharged home after prolonged hospital admission and was conservatively managed for TBAD. This case was complicated by the fact that the patient had an out-of-hospital cardiac arrest and a coronary angiogram showed severe stenosis in the obtuse marginal branch of the left circumflex artery. The patient also had new-onset atrial fibrillation, which made his clinical management very challenging. It is important to avoid unnecessary coronary intervention that can create more challenges in managing such patients.

## Introduction

Aortic dissection (AD) is a fatal but uncommon disease that requires early diagnosis and intervention to avoid fatal outcomes [1]. Chest pain is the most common presenting symptom of AD. Several conditions can mimic AD such as acute myocardial infarction (AMI), pulmonary embolism (PE), cerebrovascular accident (CVA), and acute abdominal pathology [2]. The annual incidence of acute AD in the western population ranges from 2.6 to 3.5 per 100,000 person/year and has very high mortality with up to 20% of patients dying before reaching the hospital and a further 30% dying during hospital admission [2,3]. Type B aortic dissection (TBAD) involving descending aorta accounts for 25-40% of all dissections. TBAD can be further subdivided into dissection in the descending thoracic aorta distal to the left subclavian artery and proximal to the celiac artery or dissection involving the thoracic and abdominal aorta distal to the left subclavian artery, known as DeBakey type IIIa and IIIb, respectively [4].

The mortality rate for TBAD is about 11.7% and most patients die within the first 24 hours. The clinical outcome also depends on the type of AD and complicated AD generally has a poor prognosis compared to uncomplicated AD. AD is referred to as complicated when there is a presence of aortic rupture, impending rupture, or peripheral malperfusion [4]. About two-thirds of TBAD are uncomplicated, and good blood pressure control by beta-blockers and calcium channel blockers is usually the cornerstone in managing these patients.

Malperfusion due to acute TBAD can present as a renal, spinal cord, or lower limb ischaemia [5]. TBAD patients who present with malperfusion are mostly young, male, and have high preoperative creatinine [5]. Spinal cord ischaemia due to acute TBAD is very rare and accounts for about 3% of malperfusion cases and there is a lack of effective treatment for malperfusion-induced spinal cord ischaemia. Aneurysmal degeneration of the aorta was reported in about 28.5% of patients with TBAD who were medically managed and were followed up for about 18.1 months and survival rate was reported as 77% and 56% at three and 10 years, respectively [4,6]. The operative mortality rate for patients with TBAD was reported as 13% and the 15-year survival rate was reported as 11% for patients with both complicated and non-complicated TBAD.

We present a case of a 63-year-old patient who presented with out-of-hospital cardiac arrest with the return of spontaneous circulation. The initial rhythm was pulseless electrical activity (PEA) and the patient was found to have severe stenosis of the obtuse marginal (OM) branch of the left circumflex artery on an emergency coronary angiogram. The interventional cardiologist decided not to stent the stenosed artery as this alone could not have explained his clinical presentation and a decision for percutaneous intervention was delayed till other causes for his cardiac arrest were ruled out. A computed tomography scan of the aorta and pulmonary angiogram showed TBAD and an occluded right renal artery.

## Case presentation

A 63-year-old male patient presented with witnessed out-of-hospital cardiac arrest. The initial rhythm was PEA and the patient had excellent cardiopulmonary resuscitation (CPR). He had chest pain and felt clammy and sweaty for half an hour before collapsing. The patient had CPR for 12 minutes and was administered one dose of adrenaline and return of spontaneous circulation (ROSC) was achieved after 12 minutes of CPR. The patient was slightly agitated and confused post-cardiac arrest. Past medical history was insignificant of note and he was not on any regular medication. He was a non-smoker and a social drinker.

The patient was quite hypoxic and required high flow oxygen to maintain oxygen saturation above 95%. Initial vitals were blood pressure (BP) at 87/54 mmHg, heart rate (HR) of 108 beats per minute, SpO2 at 97% on 15 litres of oxygen, and respiratory rate of 24 breaths per minute. Clinical examination was normal apart from agitation and confusion. Electrocardiogram (ECG) showed atrial fibrillation with a rapid ventricular response and ST-segment depression in lateral leads. A bedside echocardiogram showed hyperdynamic left ventricle, normal right ventricle, and mild inferior wall hypokinesia.

**Figure 1:**
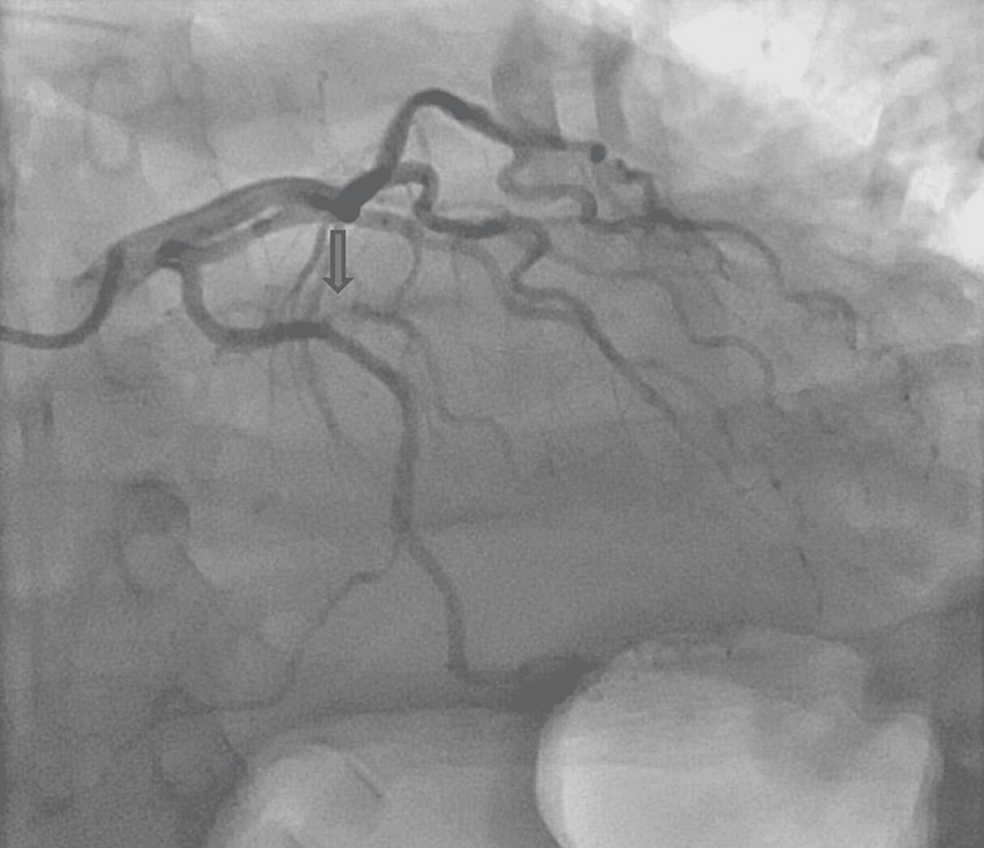
Severe stenosis at the level of ostium in the obtuse marginal branch of the left circumflex artery

The patient was intubated for his high oxygen requirement and agitation to facilitate an emergency coronary angiogram. A coronary angiogram showed severe stenosis at the ostium of the OM branch of the left circumflex artery and mild stenosis (25-49%) in the right coronary artery (RCA) (Videos 1, 2 and Figure 1).

**Video 1:** Coronary angiogram of the right coronary artery

**Video 2:** Coronary angiogram of the left coronary artery including LMS, LAD, and LCx LMS: left main stem; LAD: left anterior descending artery; LCx: left circumflex artery.

**Figure 2:**
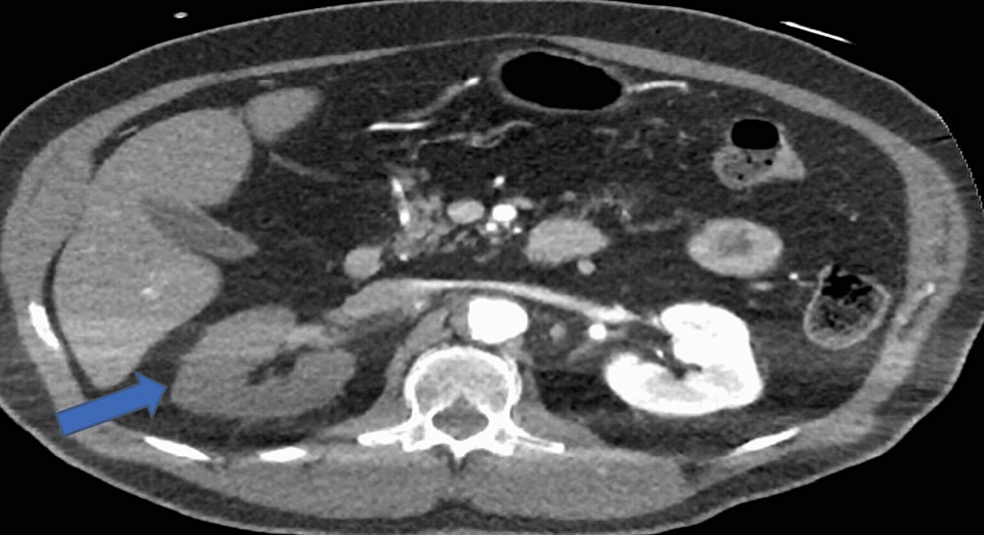
Computed tomography pulmonary angiogram showing right-sided renal malperfusion as shown by the pointed arrow

**Figure 3:**
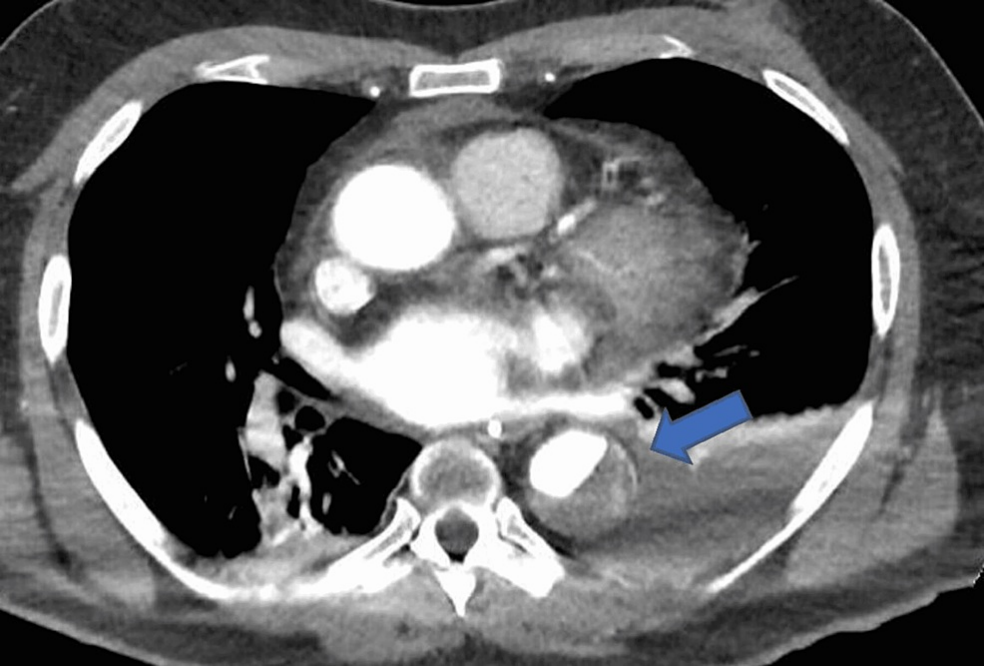
Computed tomography aortogram scan showing acute type B aortic dissection as shown by the pointed arrow

**Figure 4:**
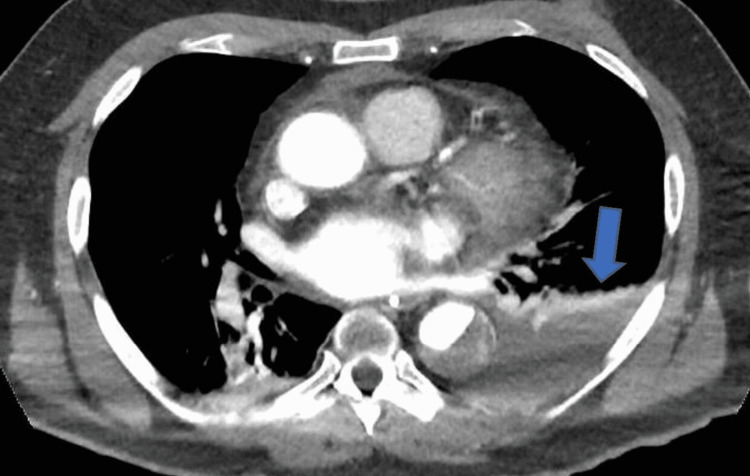
Computed tomography pulmonary angiography scan showing large left-sided haemothorax as shown by the pointed arrow

The OM branch was a small calibre artery and the interventional cardiologist decided not to proceed with percutaneous coronary intervention (PCI) to this branch immediately, as this was unlikely to be the cause of his cardiac arrest despite its severity. A computed pulmonary angiogram (CTPA) and computed angiogram of the aorta (CTAA) showed Stanford TBAD with a thrombosed false lumen and the dissection was extended from just distal to the origin of the left subclavian artery down to the level of the aortic bifurcation. The CTPA also showed large left-sided haemothorax and rib fractures (Figures 2-4).

CT of the head showed bilateral focal areas of low attenuation representing watershed infarcts secondary to hypoxic brain injury. An echocardiogram showed a normal-sized left ventricular cavity with mild concentric left ventricular hypertrophy and left ventricular ejection fraction of 55-60% and a normal right ventricle. The left atrium and aortic sinus were slightly dilated on the echocardiogram. The trend of laboratory test results is shown in Table 1.

**Table 1:** Laboratory test results trend for the patient during admission

Test	Day 1	Day 3	Day 5	Day 10	Day 15	Reference value
White cell count	20.00	12.57	13.44	22.06	17.90	3.5-11 x 10^9/L
Neutrophil	15.54	17.05	10.17	13.47	16.82	1.7-7.5 x 10^9/L
Haemoglobin	148	115	86	74	84	135-170 g/L
Platelet	252	140	168	257	309	140-400 x 10^9/L
Urea	9.5	12.6	24.1	25.3	16.8	2.9-8.2 mmol/L
Creatinine	126	243	346	200	106	66-112 umol/L
Sodium	136	135	138	147	151	135-145 mmol/L
Potassium	4.0	6.2	4.8	5.7	5.1	3.5-5.1 mmol/L
C-reactive protein	1	36	128	89	30	0-5 mg/L
Troponin	148	915	996	393	257	<14 ng/L
D-dimer	51,092	-	-	2149	1780	0-400 ng/mL
Urate levels	652	495	370	342	316	200-400 umol/L
International normalized ratio (INR)	1.0	1.2	1.0	1.0	1.1	0.9-1.12
N-terminal pro-brain natriuretic peptide (pro-BNP)	1851	1740	-	-	655	<400 ng/L

The patient was admitted to the intensive care unit (ICU) post imaging and he was reviewed by vascular surgeons. Tracheostomy was performed on the patient during ICU admission, which was then gradually weaned off. Vascular surgeons advised conservative management and the patient was commenced on labetalol infusion and moxonidine initially. The patient was not anticoagulated despite having new-onset atrial fibrillation in view of the high bleeding risk. Target blood pressure was aimed at below 120/80 mmHg and was gradually switched from labetalol infusion to oral labetalol 100 mg twice daily, amlodipine 10 mg once daily, aspirin 75 mg once daily, doxazosin 8 mg twice daily, and moxonidine 300 micrograms twice daily. Labetalol was increased to 200 mg twice daily to improve his BP control as systolic blood pressure remained > 140 mmHg. He was treated with intravenous antibiotics for aspiration pneumonia and blood cultures grew *Staphylococcus epidermidis* that was sensitive to teicoplanin and vancomycin. The patient was found to have total sensory impairment and loss of power in both lower limbs after he was extubated. Magnetic resonance imaging (MRI) of the spine showed diffusion-weighted changes within the thoracic aorta in the region of the dissection indicating underlying thrombus and focal areas of restriction were also noted within the right kidney representing infarction. These features were suggestive of long segment cord ischaemia. The patient was seen by the neurology team and was discharged to a rehabilitation centre for further rehabilitation.

The management of this patient was extremely complicated due to the fact that he had out-of-hospital cardiac arrest secondary to TBAD, had severe OM branch stenosis, new-onset atrial fibrillation, and also had renal and spinal cord ischaemia resulting in paraplegia. He also had evidence of stroke based on his head CT and anticoagulation for atrial fibrillation was not advisable in view of his dissection. In addition, the diagnosis of spinal cord ischaemia was made after he was intubated making any intervention impossible at that stage.

## Discussion

Type A aortic dissection (TAAD) and TBAD are both medical emergencies and require prompt treatment. TAAD is managed surgically while TBAD is managed medically in most cases. TBAD can be complicated by malperfusion of certain organs such as the spinal cord and kidneys and the patient may end up with permanent hemiplegia, paraplegia, or renal failure. The mortality rate for TBAD is quite high and about 20% of patients die before reaching the hospital whereas a further 30% of patients die during hospital admission. The mortality for TAAD is even higher and is reported as 1.2% per hour during the first 24-48 hours from initial presentation and 50% of patients die within the first week if left untreated [6,7]. The International Registry of Acute Aortic Dissection (IRAD) was established in 1996 and publications by IRAD have steadily increased over the last two decades to improve the management of AD [1,8].

DeBakey et al. first identified clinically distinct variants of AD as type I and II in 1965 [9]. Two primary hypotheses were proposed to explain acute AD. The first theory was based on the assumption that an initial intima tear leads to the surging of blood from the aortic lumen into the media and separates the intima from the aorta, thus creating a true and false lumen. The second theory was based on the assumption that haemorrhage occurs in the vasa vasorum in the outer portion of the media first, which then leads to intimal rupture. According to both these theories, pressure from the pulsatile blood flow extends the dissection mainly in the anterograde fashion and aortic intramural haematoma or penetrating atherosclerotic ulcer occurs in about 10-20% of acute AD cases [10-12].

Booher et al. created four distinct categories based on the timing of the AD after analysing the data from IRAD as hyperacute (<24 hours), acute (two to seven days), sub-acute (8-30 days), and chronic (>30 days) [8]. The cumulative survival showed a continuous decline throughout all four groups regardless of treatment modality [8]. The optimal therapy for AD remains unclear and few trials have been done to assess the optimal therapy for these patients. The Investigation of Stent Grafts in Aortic Dissection (INSTEAD) trial randomized patients with uncomplicated TBAD into either medical therapy or thoracic endovascular aortic repair (TEVAR) therapy [13]. It supports a complication-specific approach instead of endovascular surgery for all type B dissections and results from the trial showed that medically managed patients for TBAD had an excellent two-year survival rate [13]. The IRAD reported the rate of acute AD based on data of 463 patients to be five cases per million people in a year and two-thirds of cases were of Stanford type A [5]. Male gender and age > 60 years were reported to be risk factors in this study [5].

Acute TBAD can also present with haemiplegia or paraplegia and Estrera et al. reported that paraplegia occurred in 8.5% of these patients and 81% of these patients had bilateral lower limbs involvement [14]. The diagnosis of paraplegia can be challenging due to low incidence, and treatment can be challenging and delayed due to this, which can result in permanent paraplegia for patients. Estrera et al. studied 129 consecutive patients with confirmed acute TBAD between January 2001 and March 2002 with 33.3% of the study population being female and the mean age was 61 years [14]. They developed a protocol to manage all these patients medically and surgical intervention was considered only in case of aortic rupture, aortic expansion, malperfusion, and intractable pain. Overall hospital mortality was 10.1% for these patients and it was 19% when vascular intervention was required whereas it was 8.3% if only medical management was maintained. Acute TBAD-related complications included rupture (4.7%), stroke (4.7%), paraplegia (8.5%), bowel ischemia (7%), acute renal failure (21%), dialysis requirement (13%), and peripheral ischemia (4.7%) [14].

TEVAR is the gold standard treatment for complicated acute TBAD as it reduces the pressure of the false lumen and dilates the narrowed true lumen of the major branches [6,15,16]. Unlike, TAAD, uncomplicated TBAD generally tends to have a good prognosis if properly managed with good blood pressure control. TBAD can have fatal outcomes if it is complicated by malperfusion syndrome, drug-resistant hypertension, rupture, or impending rupture, and prompt decision-making is essential in such cases. Our patient also had complicated TBAD with right renal malperfusion, paraplegia secondary to spinal cord ischaemia, and CVA. This was further complicated by new-onset atrial fibrillation and severe stenosis of the OM branch of the left circumflex artery. A major learning point from this case report is to avoid unnecessary cardiac PCI as this can make the management of such patients very complicated and challenging.

## Conclusions

In conclusion, both TAAD and TBAD are uncommon but life-threatening complications and are associated with very high mortality and morbidity. Our patient survived acute TBAD but developed stroke, renal impairment, and paraplegia due to renal artery occlusion and spinal cord ischaemia. Patients with complicated TBAD, unfortunately, have unfavourable outcomes compared to uncomplicated TBAD patients. Blood pressure control and pain management are the cornerstones in the management of TBAD. Unnecessary intervention should be avoided in these patients, as this can complicate the management of these patients.
